# A streamlined method to determine the antibiotic resistance of plaque-forming predatory bacteria

**DOI:** 10.3389/fmicb.2025.1582371

**Published:** 2025-08-19

**Authors:** Janna Wülbern, Yvonne Carstensen, Florian Buchholz, Hinrich Schulenburg, Julia Johnke

**Affiliations:** ^1^Evolutionary Ecology and Genetics, Zoological Institute, Kiel University, Kiel, Germany; ^2^Antibiotic Resistance Group, Max-Planck-Institute for Evolutionary Biology, Ploen, Germany

**Keywords:** *Bdellovibrio*, antibiotic, plaque assay, E-tests, antibiotic resistance, BALOs, Pseudomonas aeruginosa

## Abstract

**Introduction:**

Antimicrobial resistance (AMR) is a critical global health issue caused by antibiotic overuse, leading to the rise of multi-resistant pathogens such as in bacteria of the ESKAPE group. Alternative or combination therapies, including bacteriophages and plaque-forming predatory bacteria, are being explored in response. *Bdellovibrio bacteriovorus*, a Gram-negative bacterial predator belonging to the *Bdellovibrio* and like organisms (BALOs), can kill other Gram-negative bacteria after the periplasmic invasion, including multidrug-resistant pathogens. However, a combined treatment of antibiotics and plaque-forming predatory bacteria requires the predatory bacteria to be resistant to the antibiotic. The predator’s unique growth requirements limit standardized AMR testing methods.

**Materials and methods:**

We propose a streamlined three-step protocol to measure AMR in plaque-forming predatory bacteria. It requires the (i) cultivation of a dense *Bdellovibrio* culture with a suitable prey strain, followed by (ii) a double-layered agar plaque assay using a prey strain resistant to the antibiotic of interest, and (iii) the application of E-test strips for minimum inhibitory concentration (MIC) determination. We apply the method to the commonly used strain *B. bacteriovorus* HD100. We use *P. aeruginosa* H03 as prey for MIC determination for five antibiotics.

**Results:**

Our results show consistent MICs for *B. bacteriovorus* HD100 across independent experiments. Reliable MIC determination for meropenem was limited by *P. aeruginosa* H03 susceptibility to this antibiotic. Further, we observed a positive association between MIC values and predator inoculum concentration for ceftazidime, ciprofloxacin, and gentamicin. Prolonged incubation time increased MIC values, notably for ciprofloxacin. While resistant to piperacillin, predator plaques were absent on plates with piperacillin-tazobactam combinations.

**Conclusion:**

The streamlined approach described here to determine MICs in plaque-forming predatory bacteria proves effective and robust, when using a suitable (i.e., resistant) prey. It provides a starting point for the joint study of antibiotics and plaque-forming predatory bacteria.

## 1 Introduction

Antibiotics are indispensable to our healthcare system, but over time, their overuse has led bacteria to evolve resistance. The antimicrobial resistance (AMR) crisis has been classified as a global health concern by the World Health Organization (WHO) (Michael et al., 2014). In 2017, the WHO called for new antimicrobial development for a list of pathogens and designated “priority status” to the multi-resistant ESKAPE pathogens (*Enterococcus faecium*, *Staphylococcus aureus*, *Klebsiella pneumoniae*, *Acinetobacter baumannii*, *Pseudomonas aeruginosa*, and *Enterobacter* species), which cause the majority of nosocomial infections (De Oliveira et al., 2020).

In addition to the development of new antibiotics, there are approaches to adapt the application of antibiotics, e.g., combined or sequential therapy of different antibiotics (Andersson et al., 2020; Roemhild and Schulenburg, 2019; Sanz-García et al., 2023). Others examined the possibility of using bacteriophages to defeat bacterial infections (Dufour et al., 2016; Galtier et al., 2017; Jault et al., 2019; Yen et al., 2017). However, this is accompanied by several challenges, including the innate immune response to bacteriophages and the rapid evolution of bacterial resistance to phages during therapy (Kortright et al., 2019).

Because both novel antibiotics and phage therapy face practical hurdles, a third biological option—predatory bacteria—has attracted attention. Several studies have explored the therapeutic potential of predatory bacteria, i.e.*, Bdellovibrio bacteriovorus*, with encouraging outcomes observed *in vivo* in poultry (Atterbury et al., 2011), rats (Shatzkes et al., 2016), mice (Findlay et al., 2019), and zebrafish (Willis et al., 2016).

**Figure 1:**
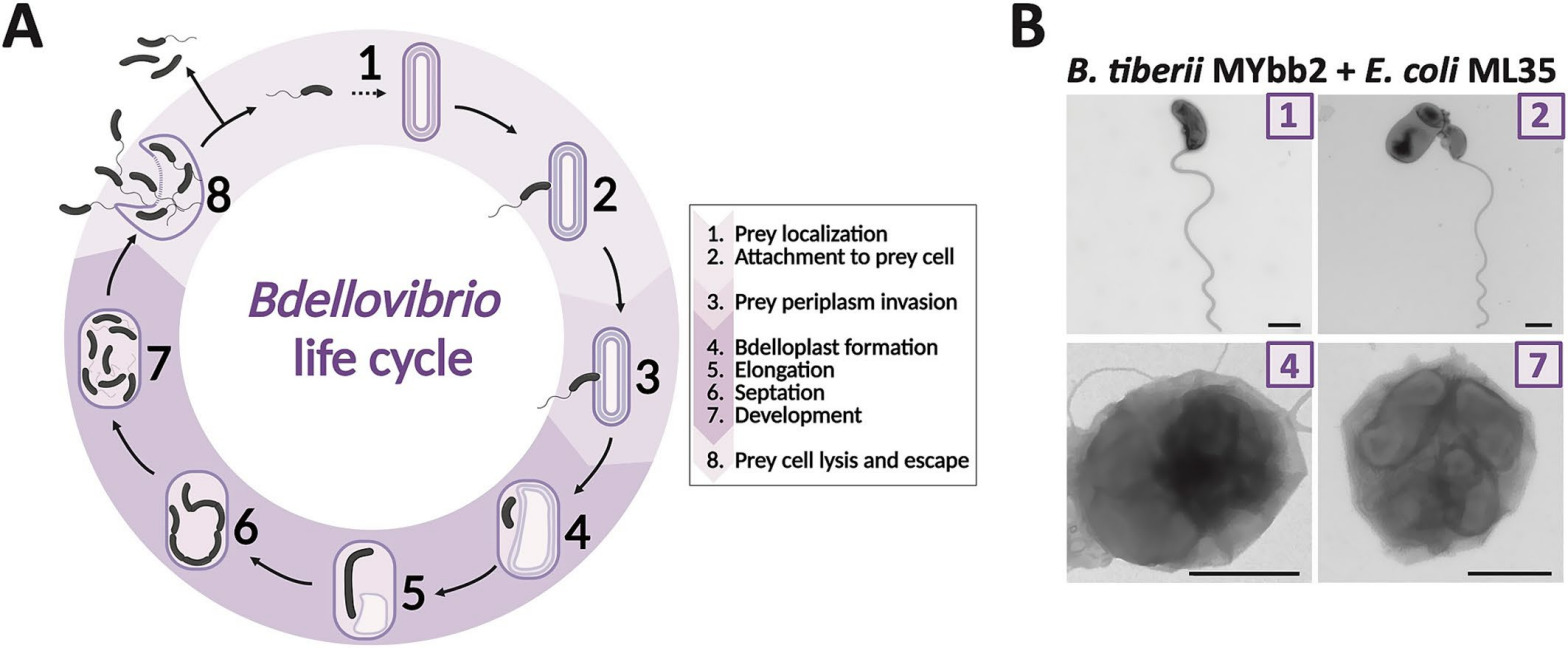
Periplasmic life cycle of *Bdellovibrio*. **(A)** In their attack phase, a free-swimming predator cell actively seeks out prey (1). Upon locating a Gram-negative cell, it attaches to the prey’s cell wall (2). Following attachment, the predator enters the prey-associated phase, invading the prey’s periplasm (3), and sealing the damaged cell wall behind them. Inside the resulting bdelloplast (4), the predator cell elongates (5), undergoes septation (6), and produces up to seven progeny cells. Once the progeny develops flagella (7), the prey cell is lysed, releasing the newly formed *Bdellovibrio* cells (8), which then seek new prey to continue the cycle. **(B)** *B. tiberii* MYbb2 with prey *E. coli* ML35 in different life cycle stages: Numbers in image corners correspond to the proposed life cycle stage in A. In stage (2), the predator is attached to an *E. coli* prey cell. Stages (4) and (7) demonstrate intraperiplasmic growth in the bdelloplast, with one or more *Bdellovibrio* cells confined within the outer membrane of a prey cell. Samples were imaged by TEM after negative staining. Scale bar is 500 nm. Life cycle illustration was created in BioRender. Wülbern (2025) https://BioRender.com/6ybxp6x, Figure based on Maher et al. (2025), modified by the authors.

*B. bacteriovorus* is a highly motile, Gram-negative obligate predator that belongs to the group of *Bdellovibrio* and like organisms (BALOs) whose members are found within the phylum *Bdellovibrionota* (genera *Bdellovibrio*, *Pseudobdellovibrio*, *Bacteriovorax*, *Peredibacter, Halobacteriovorax*, *Pseudobacteriovorax*, and *Micavibrio*). All BALOs are characterized by a biphasic life cycle. The attack phase comprises free-swimming predator cells that actively seek out prey (Figures 1A,B). Upon locating Gram-negative bacteria, they enter a prey-associated phase, which starts with BALOs attaching to the prey cells. Periplasmic predators then invade the periplasm of the prey. Sealing the damaged peptidoglycan wall behind them, they form an osmotically stable bdelloplast, establishing and growing through elongation and non-binary fission. Once flagella have developed, the bdelloplast is lysed, releasing up to seven progenies that immediately enter the attack phase (Laloux, 2020). In contrast, epibiotic predators remain attached to the outside of the prey cell while consuming its cellular content. Various *B. bacteriovorus* strains demonstrate a broad host range, including ESKAPE pathogens (Negus et al., 2017). Unlike bacteriophages, *B. bacteriovorus* rapidly hydrolyzes all genomic DNA within its prey, reducing the risk of horizontal gene transfer (Matin and Rittenberg, 1972; Monnappa et al., 2013), a key mechanism in the spread of AMR (Djordjevic et al., 2024). Further, this predator has been shown to invade and destroy biofilms (Kadouri and O’Toole, 2005; Núñez et al., 2005), which can act as protective barriers for bacteria against antibiotics (Stewart and Costerton, 2001). Further, a recent study demonstrated that *Bdellovibrio* can effectively prey on antibiotic persister *E. coli* cells, which are typically tolerant to conventional antibiotics (Matan and Jurkevitch, 2022). Importantly, *B. bacteriovorus* is non-pathogenic to human serum and cell lines *in vitro* (Baker et al., 2017; Gupta et al., 2016; Monnappa et al., 2016; Raghunathan et al., 2019), highlighting its role as a promising “living antibiotic” against multi-resistant pathogens. However, *B. bacteriovorus* does not eliminate its prey. Although the exact mechanisms of prey resistance remain to be elucidated (Das and Negus, 2024), several studies have reported environmentally induced phenotypic prey resistance, where prey cells transition into non-susceptible physiological cell states (Kadouri and O’Toole, 2005; Shemesh and Jurkevitch, 2004; Youdkes et al., 2020). This implies that while effectively reducing bacterial load, *B. bacteriovorus* cannot fully clear the infection on its own (Cavallo et al., 2021). Therefore, combining *B. bacteriovorus* with a second agent, like an antibiotic, may be necessary to effectively treat infections and prevent the spread of new AMR genes. In this regard, Im et al. (2017) showed that combining violacein with *B. bacteriovorus* HD100 reduced pathogen viability by 2,970-fold (99.96%) in mixed bacterial cultures.

To explore combinations of antibiotics and plaque-forming bacteria (such as *B. bacteriovorus*) against Gram-negative pathogens, a reliable method for determining predator antibiotic susceptibility is needed. As these predators only grow in the presence of prey and usually do not form colonies, the standard methods for determining the minimum inhibitory concentrations (MIC), such as the gradient method via E-test strips on bacterial lawns, cannot be applied.

**Figure 2:**
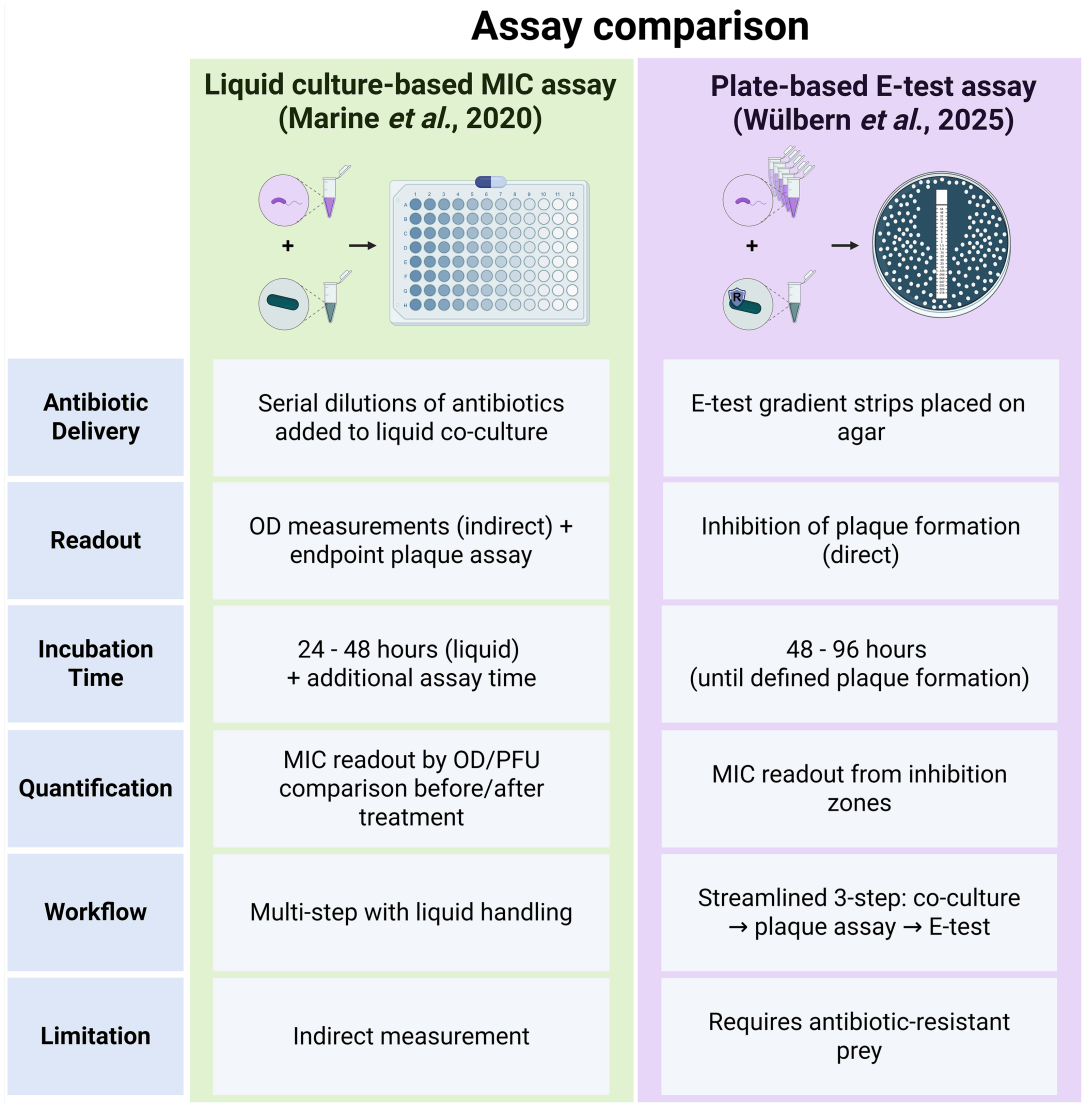
Comparison of two methods for MIC determination in plaque-forming predatory bacteria. The left panel summarizes the liquid culture-based MIC assay as described by Marine et al. (2020), which relies on indirect readouts (optical density) and multiple incubation steps. The right panel illustrates the streamlined E-test-based method introduced in this study, which directly measures inhibition of predator plaque formation on agar plates. Key differences include antibiotic delivery, readout type, incubation time, assay complexity, and methodological limitations. Created in BioRender. Wülbern (2025) https://BioRender.com/6ybxp6x.

Marine et al. (2020) proposed an assay to determine AMR in *B. bacteriovorus*. Specifically, the method requires a predator–prey co-culture in liquid medium to estimate *B. bacteriovorus* growth by measuring absorbance at 600 nm of the said co-culture with various concentrations of antibiotics. Due to their small size, *B. bacteriovorus* cells barely contribute to optical density measurements (OD_600_). Hence, measured absorbance indirectly reflects the decreasing cell density of the *E. coli* prey cells in co-cultures with *B. bacteriovorus*. This was followed by a plaque assay to verify the predator–prey ratio detected by absorbance at 600 nm (Marine et al., 2020). While this method yielded encouraging results, it involved extensive data analysis to determine the AMR profile of tested *B. bacteriovorus* strains. A key challenge in MIC testing for plaque-forming predatory bacteria remains the need for live prey, which are themselves susceptible to antibiotics and can confound results. While the Marine et al. (2020) approach is valid, MIC testing on plates with antibiotic test strips (e.g., E-tests) can simplify the process by allowing direct readout and eliminating the need for liquid culture transfers (Figure 2). To date, however, a method for MIC analysis of plaque-forming predatory bacteria on plates is not available.

This study proposes an alternative, streamlined three-step assay using E-test strips on plaque plates to determine the predator’s MIC. To ensure broad applicability across clinically relevant antibiotics, we evaluated the sensitivity of two different *Bdellovibrio* strains to antibiotics from five pharmaceutical classes, representing various mechanisms of action. We demonstrated that applying E-test strips to double-layered agar plates with a multi-resistant prey bacterium (e.g.*, P. aeruginosa* strain H03) provides reliable MIC values for all measurable antibiotics. For this approach, selecting an appropriate prey strain is critical, as sensitivity of the prey to the tested antibiotic can prevent accurate determination of the MIC for the plaque-forming bacterium.

## 2 Materials and methods

### 2.1 Resources and reagents

Bacterial strains and MIC test strips used in this study are detailed in Table 1. Table 2 lists all reagents, their respective concentrations, and preparation instructions.

**Table 1:** Key resources.

Resource	Source	Identifier
Bacterial strains		
*B. bacteriovorus* HD100	Rendulic et al. (2004)	HD100
*B. tiberii* MYbb2	Maher et al. (2025)	MYbb2
*E. coli* ML35	Buttin et al. (1956) and Casale et al. (2018)	ML35
*P. aeruginosa* PA14	Rahme et al. (1995)	PA14
*P. aeruginosa* H03	Cramer et al. (2012), Hilker et al. (2015), Tueffers et al. (2024), and Wiehlmann et al. (2007)	H03
E-tests		
Ceftazidime MIC Test Strip 0.016–256 μg/mL (CAZ)	Bestbion - LioFilchem	01B10061
Meropenem low MIC Test Strip 0.002–32 μg/mL (MRP)	Bestbion - LioFilchem	01B10148
Piperacillin MIC Test Strip 0.016–256 μg/mL (PIP)	Bestbion - LioFilchem	01B10188
Piperacillin/tazobactam (4 μg/ml) MIC Test Strip 0.016–256 μg/mL (TZP)	Bestbion - LioFilchem	01B10190
Ciprofloxacin MIC Test Strip 0.002–32 μg/mL (CIP)	Bestbion - LioFilchem	01B10082
Gentamicin low MIC Test Strip 0.016–256 μg/mL (CN)	Bestbion - LioFilchem	01B10127

Bacterial strains and resources used in this study are listed along with their source and the corresponding identifier.

**Table 2:** Table of reagents.

Reagent		Final concentration	Notes
Buffer	HEPES buffer	25 nM	Adjust to pH 7.6–7.8, after autoclaving, add 2 mM CaCl_2_ and 3 mM MgCl_2_.
Media	10% DNB (Diluted nutrient broth) agar	10% (0.8 g/L) NB 1.5% (15 g/L) BactoAgar	Adjust to pH 7.4, after autoclaving, add 2 mM CaCl_2_ and 3 mM MgCl_2_.
	10% DNB soft agar	10% (0.8 g/l) NB 0.75% (7.5 g/l) BactoAgar	Adjust to pH 7.4, after autoclaving, add 2 mM CaCl_2_ and 3 mM MgCl_2_.
	10% DNB medium	10% (0.8 g/l) NB	Adjust to pH 7.4, after autoclaving, add 2 mM CaCl_2_ and 3 mM MgCl_2_.
	LB medium	25 g/L Lysogeny broth	-

Media and buffer used in this study are listed with corresponding concentrations and preparation instructions.

### 2.2 Tested antibiotics

To determine *Bdellovibrio* AMR profiles, we utilized the multi-resistant strain *P. aeruginosa* H03 as background prey for plaque assays. We selected antibiotics from different pharmaceutical classes based on the resistance profile of H03 as determined in Tueffers et al. (2024).

### 2.3 Methods

To evaluate AMR profiles of plaque-forming predatory bacteria with the proposed method, it is necessary to (i) establish a dense culture of *Bdellovibrio* with a suitable prey strain. This culture is then used in a plaque assay (ii), using a prey strain resistant to the AB of interest, since the method relies on a background lawn of the prey to visualize lysis. And lastly, an E-test strip is added to this plaque assay plate (iii). In the following paragraphs, we will explain these steps in more detail. Thereafter, we give detailed methods used to identify factors that affect the robustness of the newly proposed method, i.e., inoculum concentration effects on MIC, and describe the statistical methods used to evaluate this effect.

*Note*: E-test-based MIC values may vary based on a variety of assay parameters, including cell density (Smith and Kirby, 2018; Udekwu et al., 2009), the growth medium, its pH, incubation time, and incubation temperature (Kowalska-Krochmal and Dudek-Wicher, 2021). To minimize variability and ensure reliable results, it is important to follow standardized protocols throughout the proposed assay.

- Cultivation of *Bdellovibrio* with a suitable prey strain

We used the standard laboratory strain *B. bacteriovorus* HD100 to establish and evaluate the robustness of the proposed assay. *Note:* To test the desired *Bdellovibrio* AMR profile, it is crucial to ensure robust bacterial growth and high cell viability for the start culture. To achieve this, we co-cultivated *Bdellovibrio* cells with the common prey strain *Escherichia coli* ML35 in DNB liquid medium for maintenance, as liquid co-cultures with *P. aeruginosa* H03 yielded comparably lower cell counts (Supplementary Figure S1). The plaque assay and E-test were then performed using the highly AB-resistant strain *P. aeruginosa* H03 as prey.

As obligate predators, *Bdellovibrio* cells require co-cultivation with prey bacteria. *E. coli* strain ML35 and *P. aeruginosa* strain H03 (Cramer et al., 2012; Hilker et al., 2015; Tueffers et al., 2024; Wiehlmann et al., 2007) were used as prey cells for liquid co-cultures and plaque assays, respectively. Prey cell overnight cultures were started from glycerol stocks (80%) and grown in 50 mL LB (Lysogeny broth) medium, continuously shaking for 16 h at 37°C and 180 rpm. Thereafter, prey cells were adjusted to an OD_600_ of 10 after being washed in 25 mM HEPES (2-[4-(2-hydroxyethyl)piperazin-1-yl] ethane sulfonic acid solution, pH 7.6–7.8, supplemented with 2 mM CaCl_2_ and 3 mM MgCl_2_) (Jurkevitch, 2012).

For *Bdellovibrio* cultivation in liquid medium, we followed the protocol of Remy et al. (2022) with minor adaptations. In short, single plaques were aspirated from double-layered DNB (10% diluted nutrient broth, supplemented with 2 mM CaCl_2_ and 3 mM MgCl_2_) agar plates, released into a sterile glass tube with 2.7 mL DNB medium with 300 μL of *E. coli* ML35 (final OD_600_ = 1), and incubated overnight shaking (180 rpm) at 28°C (Step I). After 24 h, 100 μL of the co-cultivate was transferred to fresh 2.6 mL DNB medium with 300 μL of *E. coli* ML35 (final OD_600_ = 1) and incubated overnight, shaking (180 rpm) at 28°C (Step II).

*Note:* The method described here is intended to produce a dense *Bdellovibrio* culture. To estimate culture density, we propose a visual assessment. *Bdellovibrio* predation leads to the clearing of the culture, which can be readily observed by eye. Under the microscope, using 40 × or 100 × magnification, *Bdellovibrio* cells should appear small, fast-swimming, and should constitute the majority of cells in the co-culture after incubation. Since exact cell counts are difficult to determine prior to E-test application, it is important to use multiple dilutions of the *Bdellovibrio* culture to achieve optimal conditions (details provided below).

- Plaque assay

*Note:* To assess the *Bdellovibrio* AMR profile in the proposed assay, the readout is reflected in the absence of plaque-forming units (PFUs) on double-layered agar plates. Hence, selecting a suitable prey strain is critical for establishing a reliable background to differentiate between areas where *Bdellovibrio* was able to predate and form PFUs and those where it was inhibited by the antibiotic tested. In this study, we chose *P. aeruginosa* H03, a multi-resistant and suitable prey strain for *B. bacteriovorus* HD100. Sensitivity of the prey strain to specific antibiotics may make it necessary to switch to a different strain that is then resistant to such specific antibiotics.

**Figure 3:**
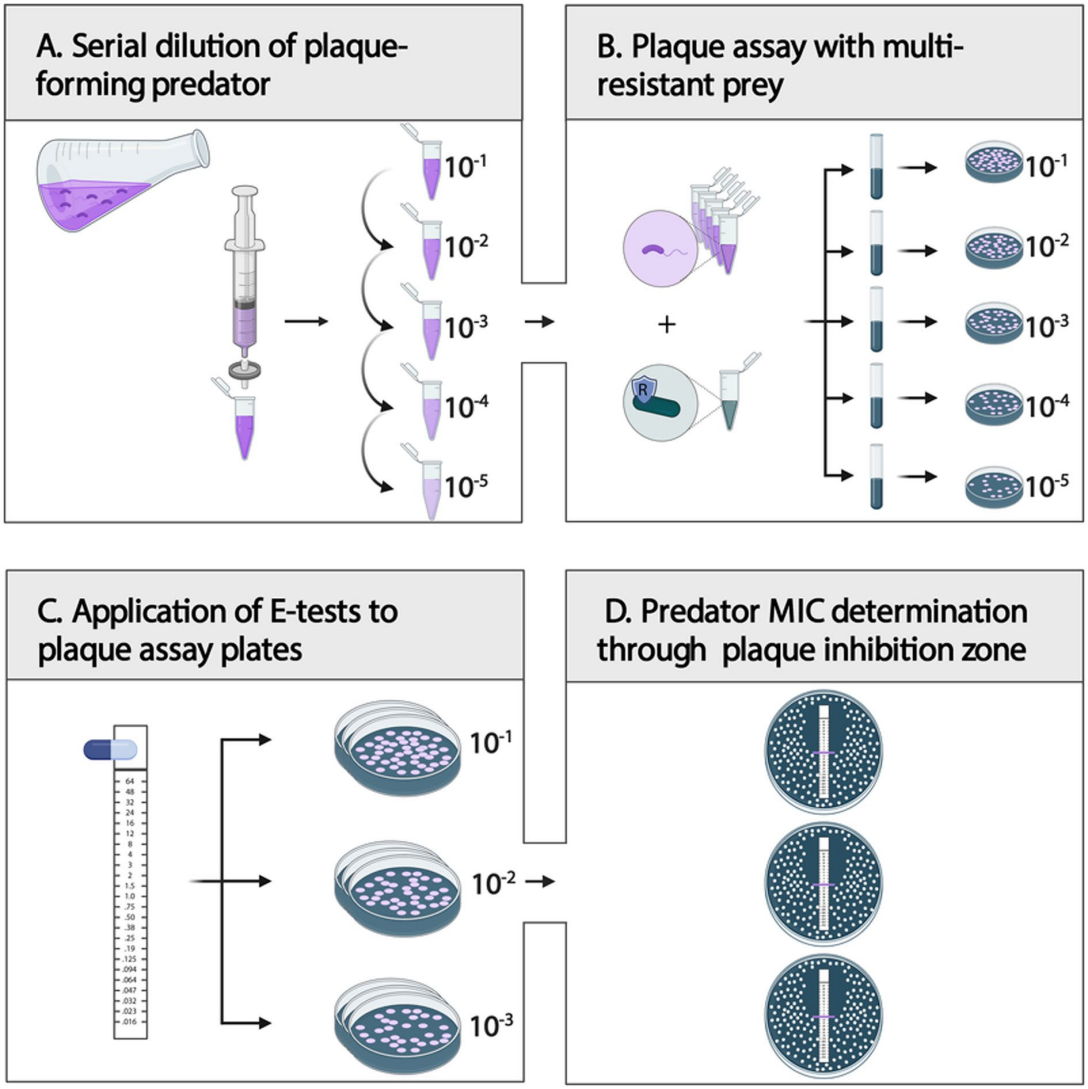
Step-by-step guide for plaque-forming predator MIC determination. **(A)** After double-filtration (0.45 μm) of a predator–prey co-culture, prepare a ten-fold serial dilution (10^0^–10^−5^) of the plaque-forming predatory bacteria. **(B)** Combine respective predator dilution with a multi-resistant prey of choice in DNB soft-agar and plate for plaque assay. Dry plates for 30 min. **(C)**: Place E-test strips onto the soft agar layer of plaque assay plates of three replicates, and dry plates for another 10 min. **(D)**: After incubation of inverted plates at 28°C for 4–7 days, evaluate the MIC of the plaque-forming predatory bacteria. Created in BioRender. Wülbern (2025) https://BioRender.com/6ybxp6x.

*Note*: For reliable readout, ensuring good quality plaque assay plates is important. Hence, it is important to pour the molten DNB soft agar evenly and to be sure to dry the plaque plates for at least 30 min, before submitting E-test strips to the plates.

Before each plaque assay, Step I and Step II *Bdellovibrio* cells were pooled for higher cell yield and separated from the remaining ML35 prey cells and debris by double-filtration through 0.45 μm syringe filters (Figure 3A). The successful removal of prey cells and debris should be confirmed microscopically. Cells were concentrated by centrifugation for 10 min at 4000 rpm. *Bdellovibrio* cell pellets were then resuspended in 1 mL 25 mM HEPES. A ten-fold serial dilution of the resuspended *Bdellovibrio* cells (10^0^–10^−5^) was prepared in 25 mM HEPES. Double-layered DNB agar plates were prepared according to standard protocol (Jurkevitch, 2012). In detail, prepared DNB agar plates (1.5% agarose) served as a nutrient layer. For the top DNB soft agar layer (0.75% agarose), 300 μL *P. aeruginosa* H03 prey cells (final OD_600_ = 0.6) and 100 μL concentrated *Bdellovibrio* cells were added to 5 mL molten DNB soft agar (58°C), mixed, and poured over the DNB agar plate, yielding a ~ 3 mm layer. Double-layered plates were allowed to dry for 30 min, before proceeding (Figure 3B).

- Determination of *Bdellovibrio* MIC by E-test

For colony-forming bacteria, the MIC of an antimicrobial is routinely assessed using the gradient method (Kadeřábková et al., 2024). For this, E-test strips containing a predefined gradient of antibiotic concentrations are applied to inoculated agar plates. The MIC value is read as the intercept of the inhibition ellipse edge. The present study employed E-test strips (LioFilchem, Italy) to double-layered plaque assay plates seeded with *P. aeruginosa* H03 and a *Bdellovibrio* strain. In detail, following the preparation of dry plaque assay plates, sterile, fully thawed E-tests (RT) were placed onto the soft agar layer of the respective dilution plates (Figure 3C). This step was performed in triplicates.

*Note*: Determining optimal predator dilutions used in the plaque assay is essential to avoid complete prey lawn clearance at low dilutions or low plaque density at high dilutions, both of which hinder accurate quantification. Pre-evaluating optimal dilutions minimizes costs by preventing repeated trials with E-tests (see “Quantification of inoculum concentration”).

After E-test placement, plates were dried for 10 min and then sealed with parafilm. Inverted plates were incubated at 28°C for four to 7 days.

*Note*: When using MIC E-test strips for colony-forming bacteria, the standard incubation time ranges between 16 and 48 h at 37°C. However, these incubation periods are not applicable to plaque-forming predatory bacteria. Depending on parameters such as the prey used in assays, the incubation time for *Bdellovibrio* can range between 48 and 96 h before the appearance of the first plaques. Antibiotics from E-test strips typically diffuse into the agar and remain stable for extended periods—up to 10 days with the LioFilchem E-test strips used in this study (according to manufacturer information). To ensure accurate results during prolonged incubation, care must be taken to prevent the plates from drying out. We also tested for antibiotic degradation during prolonged incubation and found no evidence of such degradation after up to 8 days (Supplementary Figure S2; detailed methodology is provided below). Once plaques appear, plates should be incubated for a predetermined period to ensure sufficiently high cell densities for accurate MIC determination and reproducibility across experiments.

Plaque formation was monitored in 24-h intervals, and MICs were determined once plaques were sufficiently large, yet distinguishable and dense to allow for accurate MIC evaluation (Figure 3D). The lowest antibiotic concentration at which predator plaque growth was inhibited completely was interpreted as the intercept of the inhibition zone.

*Note*: We recommend consulting the Liofilchem^®^ MIC Test Strip Photographic Guide for adequate MIC value interpretation.^1^

### 2.4 Quantification of inoculum concentration

To determine the inoculum concentration of *Bdellovibrio* cells, we used *E. coli* ML35 as prey, as preliminary experiments yielded higher predation efficiency with this strain compared to *P. aeruginosa* H03 (Supplementary Figure S1). We determine the inoculum concentration using plaque-forming units (PFUs)/ml of *Bdellovibrio* culture. For accurate PFU determination, it is essential to use plates where plaques are both distinguishable and sufficiently dense (on 9 cm plates: app. PFU > 30). Therefore, higher dilutions (10^−4^ and 10^−5^) from the ten-fold serial dilutions were utilized for cell density determination. Optimal cell density dilutions may need to be adjusted based on respective conditions, prey, and the plaque-forming predatory bacterial strain used. Double-layered DNB agar plates were prepared according to standard protocol (Jurkevitch, 2012) and as described before (see ii. Plaque assay). Cell numbers (PFU/ml) were determined by counting the total number of plaques per plate and adjusting the value based on the dilution factor and total plated volume.

### 2.5 Statistical analysis

Data analysis was performed using R (v. 4.3.2, R Core Team, 2023). Statistical analysis was performed using the “lme4” package (v.1.1.35.5, Bates et al., 2015). Plotting was performed using the “ggplot2” package (v.3.5.1, Wickham, 2016). A linear mixed model with the formula log2(MIC) ~ log2(PFU/ml) + (1|biological replicate) was used for the antibiotics ciprofloxacin (CIP) and gentamicin (CN) to examine the relationship between MIC values and PFU counts, but not for the other antibiotics as they were either lacking MIC variability (PIP/TAZ) or as they showed no linear trend (CFZ). To account for dependencies within the technical replicates associated with the same biological replicate, the biological replicate was included as a random factor. MIC and PFU/ml values were log-transformed to meet the assumptions of normality for the residuals.

### 2.6 Test for antibiotic degradation over prolonged incubation times

To assess potential antibiotic degradation during prolonged incubation, a standard E-test assay was performed using three biological replicate cultures of the *P. aeruginosa* reference strain PA14 for all antibiotics tested (CIP, CN, CAZ, TZP), following the manufacturer’s protocol (LioFilchem). Incubation was extended to 8 days, and MICs were recorded at both 24 h and 8 days.

### 2.7 Validation of MIC values obtained from E-tests

MICs for *B. bacteriovorus* HD100 and gentamycin, as determined via E-test, were validated using an E-test-independent method. Gentamycin was added at varying concentrations (0.032, 0.053, 0.106, 0.2, and 0.4 μg/mL) to DNB agar plates and DNB soft agar, followed by a standard plaque assay with three biological replicate cultures of *B. bacteriovorus* HD100 as described earlier. As in the E-test, the multi-resistant *P. aeruginosa* strain H03 was used as prey. To account for the observed inoculum effect, predator inoculum size was simultaneously determined by performing a parallel plaque assay without antibiotics, using *E. coli* ML35 as prey. All plates were incubated at 28°C for 7 days.

## 3 Results

This study aimed to establish a reliable method to test the antimicrobial resistance (AMR) of plaque-forming predatory bacteria such as *Bdellovibrio bacteriovorus*. We also examined the impact of two additional parameters, i.e., incubation duration and inoculum concentration.

### 3.1 Robust readouts for MIC values for plaque-forming predatory bacteria

**Figure 4:**
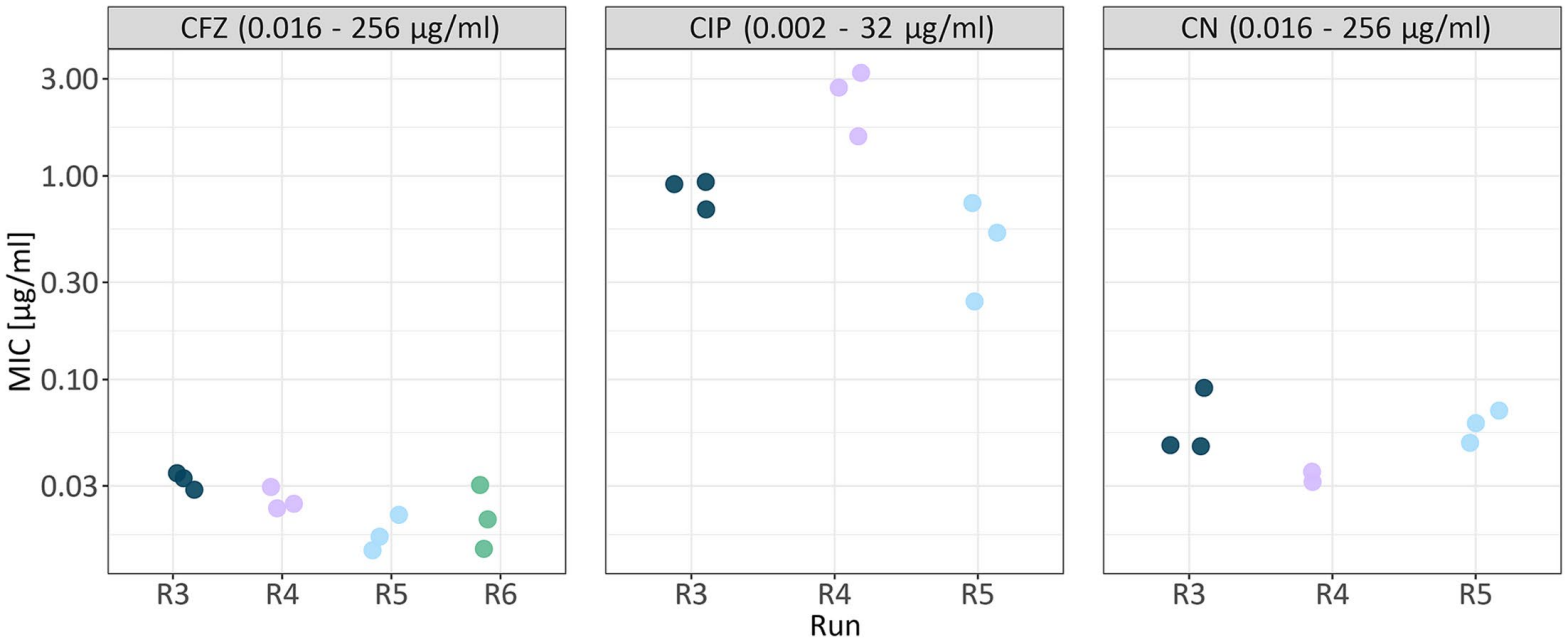
MIC values of technical replicates across runs for the most comprehensive dilution dataset. Only MIC values determined at a dilution of 10^6^ PFU/ml are shown. Each antibiotic (CFZ, ceftazidime; CIP, ciprofloxacin; CN, gentamicin) is displayed in a separate panel. Data points are colored by biological replicate (run), and for each run, technical replicates are shown. Values given in the boxes on top of the plot indicate the antibiotic concentration range of the respective MIC test strip.

We evaluated the susceptibility of *B. bacteriovorus* HD100 to five antibiotics of various pharmaceutical classes: ceftazidime (CFZ), meropenem (MRP), piperacillin (PIP), piperacillin/tazobactam (TZP), ciprofloxacin (CIP), and gentamicin (CN) (Table 3). The assay was performed in five independent runs, each using a distinct *Bdellovibrio* culture and varying initial inoculum concentrations based on the respective culture densities. Across these runs, minimal variation in MIC values was observed among technical replicates (Table 4; Figure 4). Dilutions with an inoculum concentration in the range of 10^6^ PFU/ml provided the most comprehensive data across antibiotics (Table 4; Supplementary Table S1). Supplementary Table S1 includes a detailed overview of individual MIC values per run and precise PFU/ml concentrations.

**Table 3:** Antibiotics used for MIC determination.

Antibiotic	Pharmaceutical class	Mechanism of action
Ceftazidime (CFZ)	Cephalosporin	Cell wall synthesis
Meropenem (MRP)	Carbapenem	
Piperacillin (PIP)	Penicillin	
Piperacillin/ tazobactam (TZP)	Penicillin/ β-lactamase inhibitor	
Ciprofloxacin (CIP)	Quinolone	DNA synthesis
Gentamicin (CN)	Aminoglycoside	Protein synthesis

Antibiotics from different pharmaceutical classes are listed along with their mechanism of action in bacteria.

**Table 4:** Mean MIC values for *B. bacteriovorus* HD100 in five independent runs.

	Run	Mean MIC ± SE [μg/ml]			
		10^4^ PFU/ml	10^5^ PFU/ml	10^6^ PFU/ml	10^7^ PFU/ml
Ceftazidime (CFZ)	R1		0.053 ± 0.011		
	R3			0.032 ± 0.000	
	R4			0.026 ± 0.003	
	R5			0.018 ± 0.002	0.084 ± 0.020
	R6			0.024 ± 0.005	
	mean		**0.053 ± 0.011**	**0.025 ± 0.002**	**0.084 ± 0.020**
Meropenem (MRP)	R1	nd	nd		
Piperacillin^a^ (PIP)	R1	≥ 256.000 ± 0.000	≥ 256.000 ± 0.000		
Piperacillin/ tazobactam (TZP)^b^	R1	≤ 0.016 ± 0.000	≤ 0.016 ± 0.000		
	R2	≤ 0.016 ± 0.000	≤ 0.016 ± 0.000		
	R3		≤ 0.016 ± 0.000	≤ 0.016 ± 0.000	
	R4		≤ 0.016 ± 0.000	≤ 0.016 ± 0.000	
	R5			≤ 0.016 ± 0.000	≤ 0.016 ± 0.000
	mean		**≤ 0.016 ± 0.000**	**≤ 0.016 ± 0.000**	**≤ 0.016 ± 0.000**
Ciprofloxacin (CIP)	R1	0.230 ± 0.020	0.460 ± 0.040		
	R2	0.583 ± 0.083	1.000 ± 0.500		
	R3		0.667 ± 0.167	0.917 ± 0.083	
	R4		1.500 ± 0.500	2.500 ± 0.500	
	R5			0.500 ± 0.144	2.333 ± 0.833
	mean	**0.407 ± 0.088**	**0.898 ± 0.191**	**1.306 ± 0.340**	**2.333 ± 0.833**
Gentamicin (CN)	R1		0.043 ± 0.011		
	R2		0.026 ± 0.010		
	R3			0.063 ± 0.016	
	R4		0.028 ± 0.002	0.032 ± 0.000	
	R5			0.058 ± 0.006	0.147 ± 0.022
	mean		**0.032 ± 0.005**	**0.053 ± 0.007**	**0.147 ± 0.022**

a Values for highest MIC intercept available for MIC Test Strip 0.016–256 μg/mL (PIP), minimal plaque inhibition was observed.

b Values for lowest MIC intercept available for MIC Test Strip 0.016–256 μg/mL (TZP), no plaque formation was observed.

MIC values are expressed as mean MIC ± standard error (SE) of technical replicates (n = 3) per run/biological replicate (R1-R6) or mean across biological replicates (in bold). MIC values were determined on plates of different inoculum concentrations, values were rounded for comparison. For meropenem (MRP), MIC values were not determinable (nd).

The inhibition of *P. aeruginosa* growth at elevated meropenem concentrations prevented reliable MIC determination for *B. bacteriovorus* HD100 with this antibiotic. Across all runs, plaque formation was observed at the highest antibiotic concentrations on *B. bacteriovorus* strain HD100 plates with E-test strips for piperacillin, indicating resistance to this *β*-lactam antibiotic (MIC ≥ 256 μg/mL). In contrast, adding a β-lactamase inhibitor, e.g., tazobactam, to render susceptibility (Cheng et al., 2017), led to no plaque formation, indicating that the MIC is below the lowest antibiotic concentration tested, ≤ 0.016 μg/mL.

We validated the observed MIC for gentamycin using an E-test-independent method by incorporating gentamycin directly into the agar and soft agar prior to performing a plaque assay. Using a *B. bacteriovorus* HD100 inoculum of approximately 10^8^ PFU/mL, we determined MIC values of 0.2 μg/mL in two of three biological replicates, and 0.053 μg/mL in the remaining replicate. These MIC values align with those obtained from E-tests conducted with similar inoculum densities (e.g., 0.147 μg/mL for 10^7^ PFU/mL).

In summary, the proposed assay produced robust MIC values for all considered antibiotics, in all cases dependent on the availability of a suitable (i.e., resistant) prey strain.

### 3.2 Relationship between MIC values and predator inoculum concentration

For colony-forming bacteria, several studies have shown a direct relationship between MIC values and inoculum density (inoculum effect) (García-Rivera et al., 2024; Smith and Kirby, 2018). In this context, we aimed to test different inoculum concentrations for *B. bacteriovorus* HD100 by applying E-test strips to different dilutions of the same biological replicates (Table 4).

**Figure 5:**
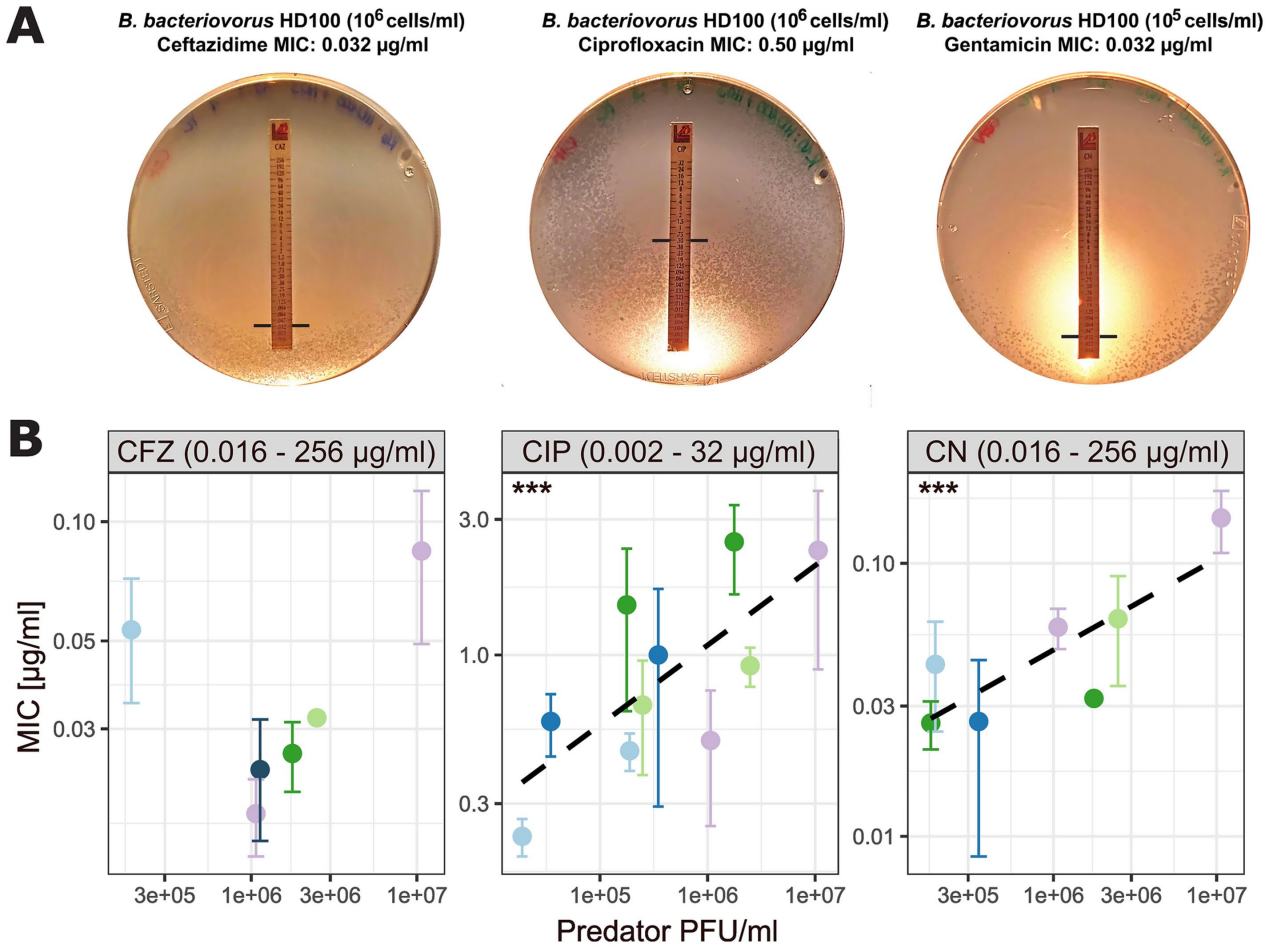
Relationship between predator inoculum concentration and MIC values. **(A)** Exemplary images of DNB agar plates showing *B. bacteriovorus* plaques with *P. aeruginosa* H03 as background lawn and E-test stripes. Black lines represent predator MIC intercept. **(B)** Association between MIC and predator inoculum concentration (measured as PFU/ml). Each antibiotic (CFZ, ceftazidime; CIP, ciprofloxacin; CN, gentamicin) is displayed in a separate panel with individual x- and y-axes. Black dashed lines for CIP and CN represent linear regression lines fitted to the data. Asterisks indicate *p*-values as calculated using a linear mixed model, with biological replicate as random variable to account for dependencies (Supplementary Table S2). Data points (colored by biological replicate) represent the mean MIC value of three technical replicates for biological replicates, with variability indicated by SD bars. Values given in the boxes on top of the plot indicate the antibiotic concentration range of the respective MIC test strip.

We tested for a linear association between mean MIC values and inoculum concentration for ciprofloxacin (linear mixed model, *p =* 9.54e-05), and gentamicin (linear mixed model, *p =* 0.000654), but not ceftazidime (Figure 5; Supplementary Table S2; Supplementary Figure S3). Interestingly, for ceftazidime, we noted higher MIC values at inoculum concentrations in the range of 10^5^ PFU/ml and 10^7^ PFU/ml compared to 10^6^ PFU/ml indicating no linear relationship. Especially for sensitive cells, E-test results may vary by 1 or 2 steps in the readable MIC between replicates due to a very small antibiotic gradient. However, for these dilutions data were obtained in one run each, showing the need for replication. In contrast, we saw little variation across four independent runs for inoculum concentrations of 10^6^ PFU/ml (Figure 5; Table 4).

### 3.3 Relationship between MIC values and incubation time

**Figure 6:**
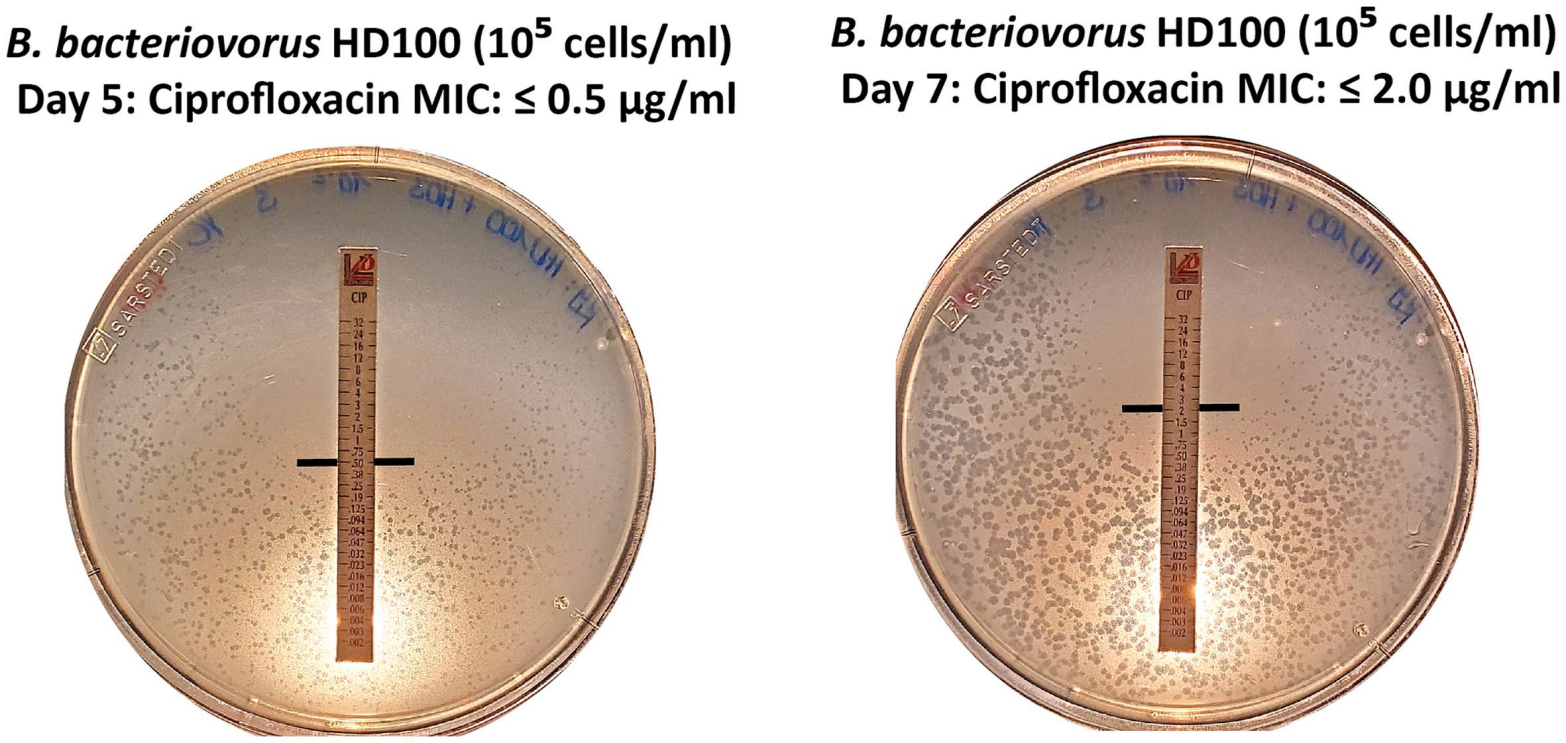
Extended incubation by 48 h yields a 4-fold increase in MIC value. For *B. bacteriovorus* HD100, the MIC intercept was determined at 0.5 μg/mL after five days and increased to 2 μg/mL after 7 days for ciprofloxacin.

First plaques of *B. bacteriovorus* HD100 appeared after four to 7 days. Plates were then incubated for another 48–72 h before MIC determination. In some cases, we observed small, scattered plaques in the periphery of inhibition zones. This may result from a reduced predator’s predation efficiency or, in general, slower growth due to increasing inhibitory concentrations. They can also be an indication of *de novo* resistant mutants, as they arise from a single cell, they will inherently be smaller. In cases where small, scattered plaques obscured the inhibition zone, the incubation time was extended in 24-h intervals until a clear MIC intercept could be reliably determined. Notably, we observed that longer incubation times influenced MIC values. Specifically, for ciprofloxacin, where the initial MIC was 0.5 μg/mL, an additional incubation time of 48 h yielded a four-fold increased MIC intercept (MIC = 2 μg/mL) (Figure 6).

### 3.4 Susceptible prey limits MIC determination for plaque-forming predatory bacteria

**Figure 7:**
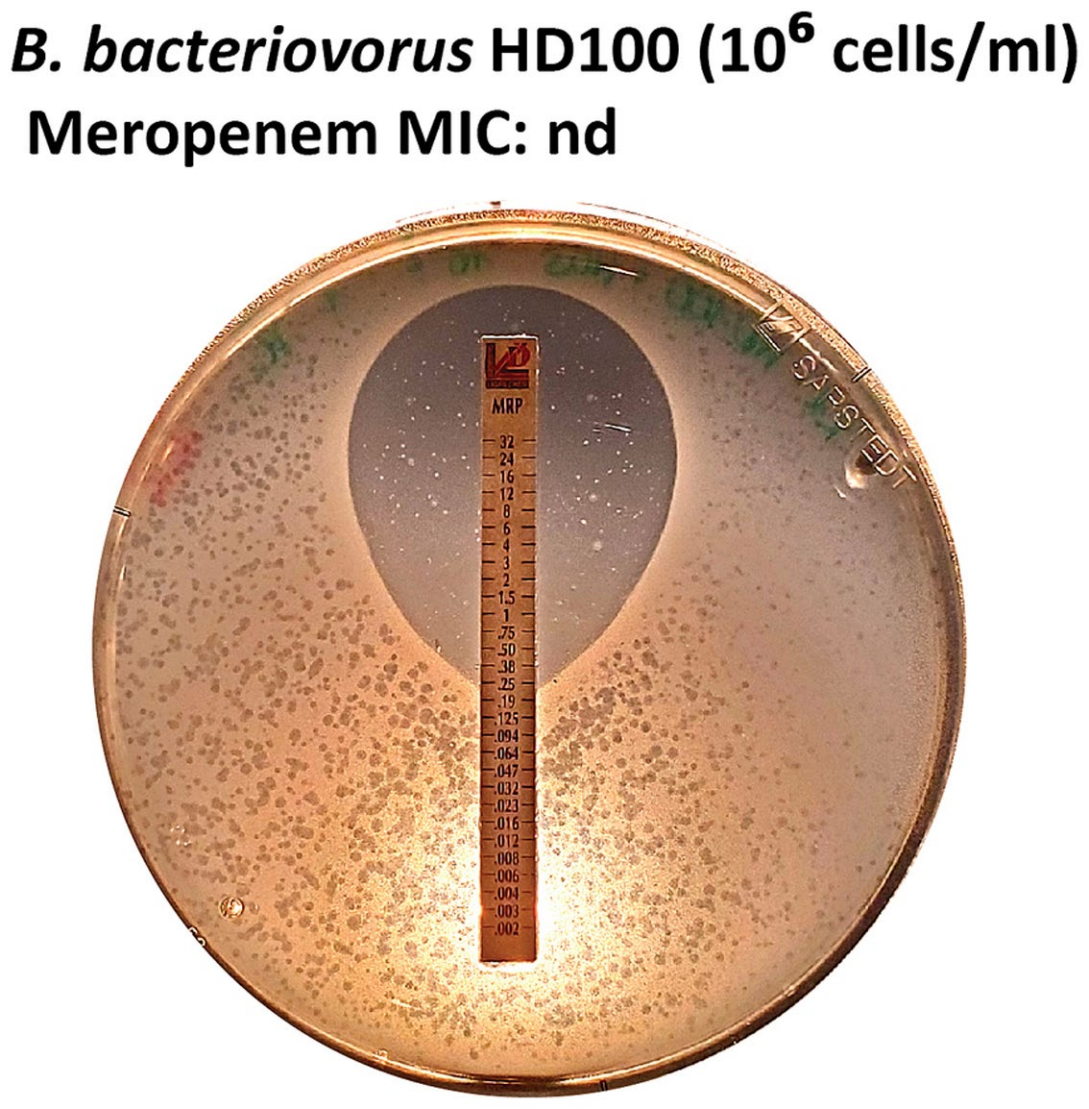
MIC not determinable for meropenem E-tests. The growth of *P. aeruginosa* H03 was inhibited at antibiotic concentrations > 0.25 μg/mL, while *B. bacteriovorus* HD100 formed plaques around the inhibition zone.

Meropenem is a carbapenem antibiotic frequently employed to treat bacteria resistant to other *β*-lactam antibiotics, such as the multidrug-resistant *P. aeruginosa* strain H03. This strain has been previously reported to exhibit susceptibility to meropenem at elevated concentrations (Tueffers et al., 2024). In line with this, the growth of *P. aeruginosa* H03 was visibly inhibited at higher antibiotic concentrations (> 0.25 μg/mL). Notably, *B. bacteriovorus* HD100 formed plaques throughout the lawn of *P. aeruginosa* H03. Therefore, a clear MIC intercept for *B. bacteriovorus* could not be determined using *P. aeruginosa* H03 (Figure 7).

### 3.5 Challenges and suggestions

The proposed assay shows great potential for determining MIC values for plaque-forming predatory bacteria when accounting for a few challenges (Table 5). Small variations in determined MIC values were linked to predator inoculum concentration and plaque plate incubation time and are further discussed in the next sections.

**Table 5:** Challenges and suggestions.

Challenge	Suggestion
Low predator inoculum concentration	Consider pooling Step I (24 h) and Step II (48 h) of predator–prey co-cultures to achieve higher cell density or prepare multiple Step II cultures from Step I cultures and pool.
High predator inoculum concentration	High plaque density can prevent accurate MIC readings. We recommend selecting an inoculum concentration that allows clear differentiation of individual plaques while distinguishing potential satellite plaques from the bacterial lawn.
Unusual plaque formation	Be sure to dry the plaque plates for at least 10 min before submitting E-test strips to the plates.
MIC value variation between replicates	Normalize predator cell counts before plaque assays. We recommend a minimum inoculum concentration of 10^5^ cells/ml for predators with similar plaque size.
MIC value variation with extended incubation time	Predefine an incubation period after the appearance of first plaques until MIC determination. We recommend 48 h. Prolonged incubation may result in microcolonies within the inhibition zone, likely due to resistance evolution.
No or incomplete background prey lawn observed on plaque assay plate	The prey strain used is sensitive to the antibiotic. Select an alternative strain that is both susceptible to predation and resistant to the antibiotic.

## 4 Discussion

### 4.1 E-tests on plaque assay plates are a suitable method to determine the AMR of plaque-forming predatory bacteria

In this study, we propose a streamlined method to determine the AMR in plaque-forming predatory bacteria. We show that prey choice, inoculum density, and experimental conditions affect the determination of AMR in *Bdellovibrio*.

In detail, we used *E. coli* ML35 as prey for *Bdellovibrio* inoculum concentration determination, as plates seeded with this prey yielded higher numbers of *Bdellovibrio* plaques than those seeded with *P. aeruginosa* H03. This difference may be linked to the production of various antimicrobial compounds to evade predation. For instance, like many bacteria utilizing the quorum sensing (QS) system, *P. aeruginosa* produces diffusible signaling factors (DSF) (Twomey et al., 2012), that can delay predation and compromise membrane integrity in *B. bacteriovorus* (Dwidar et al., 2021). A recent study showed that the production of another QS molecule, 2-heptyl-3hydroxy-4(1H)-quinolone (PQS), was toxic to *B. bacteriovorus* (Hoshiko et al., 2021). Additionally, *P. aeruginosa* synthesizes toxic compounds such as pyocyanin, a phenazine pigment, and cyanides, which have been shown to reduce *B. bacteriovorus* motility rates and inhibit its predatory activity (Mun et al., 2017). Despite this potential multi-layered line of defense, predation through *B. bacteriovorus* is not inhibited, yet decreased for *P. aeruginosa* compared to *E. coli* (Supplementary Figure S1). While the effect of pyocyanin on *Bdellovibrio* motility is unlikely to influence *Bdellovibrio* MIC, intraperiplasmic *Bdellovibrio* cells may be affected. Mun et al. (2017) demonstrated that bdelloplasts exposed to cyanides exhibit inhibited development, making them likely less metabolically active and, hence, potentially taking in fewer antibiotics that require active transport.

Beyond antimicrobial defenses, inoculum density also impacts predation dynamics. Several studies link inoculum concentration to MIC values (inoculum effect) in colony-forming bacteria (García-Rivera et al., 2024; Smith and Kirby, 2018). Our findings indicate a similar effect in plaque-forming predatory bacteria, underscoring the need for standardized inoculum densities for reliable MIC results. For consistent, reproducible MIC readouts, it is recommended to use a standardized optical density for colony-forming bacteria when applying MIC E-tests. Accordingly, we strongly recommend testing respective plaque-forming predatory bacteria using standardized cell densities, for instance by fluorescence-based inoculum quantification, as suggested by Remy et al. (2022), or by PFU/ml determination using plaque assays. Ideally, the standardized cell densities should align with the requirements of subsequent experimental assays for which the MICs were determined.

Further, when selecting a prey strain, it is crucial to consider prey susceptibility to applied antibiotics, which can affect MIC determination for plaque-forming predatory bacteria, as seen for meropenem in this study, highlighting the importance of careful prey choice. *P. aeruginosa* strain H03 has been previously reported to exhibit susceptibility to meropenem at elevated concentrations (Tueffers et al., 2024) in line with the results of our study (> 0.25 μg/mL, Figure 7). In turn, meropenem was excluded from further testing, as no definite MIC intercept could be determined for the plaque-forming predatory bacteria. Prey strains should be selected for resistance to the antibiotics being tested, e.g., strains resistant to meropenem when assessing meropenem resistance in plaque-forming predatory bacteria. Representative *Pseudomonas aeruginosa* strain panels may be consulted to identify suitable candidates (Tueffers et al., 2024). One of the advantages of our proposed method is that this limitation is easily detectable, as prey lawn inhibition can be directly observed on double agar plates.

Importantly, prey cells may release enzymes that modify or degrade antibiotics, potentially leading to an underestimation of MIC values for the plaque-forming predatory bacteria applied. Therefore, we advise using multi-resistant prey bacteria, for which resistance does not rely on secreted enzymes, but on other resistance mechanisms (e.g., target modification or efflux). In addition, incubation conditions are crucial, particularly due to the extended periods required for plaque formation. Prolonged incubation can impact antibiotic stability, independent of prey activity (Lallemand et al., 2016). For instance, *P. aeruginosa* H03 encodes genes for aminoglycoside-modifying enzymes (aph(3′)-II, aph(6)-Ic, and aac(6’)Ib4) that may lower gentamicin concentration over prolonged exposure (Tueffers et al., 2024) and thus lead to an overestimation of the predator MIC value. Additionally, extended incubation under selective pressure increases the likelihood of resistant mutants. *P. aeruginosa* produces bactericidal compounds such as pyocyanin, DSF, and PQS (Hoshiko et al., 2021; Twomey et al., 2012), which may select for resistant *Bdellovibrio* cells. Hence, small, scattered plaques observed in the periphery of the inhibition zone should be interpreted cautiously. Plaques occurring at the edge of the inhibition zone after longer incubation may be more resistant cells. We observed these cases only for the drug ciprofloxacin which is a known SOS-response inducer that can lead to hypermutation, suggesting that these plaques are indeed resistant BALOs (Crane et al., 2021). In general, MICs determined with these assays should always be confirmed within agar dilution if precise MICs are necessary. We recommend to disregard microcolonies within the inhibition zone as these are likely resistant mutants and determine the MIC on the edge of the ellipse after a standardized incubation period.

### 4.2 *Bdellovibrio* AMR profiles reveal ciprofloxacin to be a suitable candidate for combined therapy

After establishing the robustness of the method, we applied it in a preliminary test on *Bdellovibrio tiberii* MYbb2, a close relative of *B. bacteriovorus* HD100 (Maher et al., 2025). Although only tested in a single biological replicate, MIC values for MYbb2 (CFZ: 0.021 μg/mL, TZP: ≤ 0.016 μg/mL, CIP: 1.667 μg/mL, CN: 0.032 μg/mL) were within the range observed for HD100. These initial results suggest that the method may be applicable to related species, though further testing is required to confirm reproducibility and species-specific differences. Both tested strains exhibit high resistance to ciprofloxacin while showing high susceptibility to piperacillin/tazobactam. Similarly, a previous study on the antibiotic resistance of another *Bdellovibrio* species, the epibiotic *B. exovorus* JSS, reported high resistance to ciprofloxacin. Interestingly, *B. exovorus* JSS presented higher resistance to piperacillin/tazobactam than to gentamicin (Pasternak et al., 2014). This indicates that distinct *Bdellovibrio* species have distinct susceptibility patterns. More information on the different species’ AMR profiles could provide valuable insights for effective combination therapies of plaque-forming predatory bacteria and antibiotics. Additionally, the proposed assay can be used to identify clinical breakpoints for *Bdellovibrio*. These thresholds, defined by the European Committee of Antimicrobial Susceptibility Testing (EUCAST), determine antibiotic resistance and are crucial in the context of combination therapy. We want to emphasize that the here proposed assay should also be adapted to other gradient-based MIC methods, such as the disc diffusion assay. While they do not yield precise MIC values, which are critical for experimental assays, they may offer a cost-effective alternative for high-throughput screening. Ideally, a suitable antibiotic for combined therapy would be one for which the pathogen is susceptible within clinical dosing limits but against which *B. bacteriovorus* is resistant.

In this context, piperacillin might be an interesting candidate antibiotic for combination therapy, as *B. bacteriovorus* HD100 demonstrated full resistance at the highest concentrations. However, if this resistance is mediated by excreted *β*-lactamases, as indicated by the loss of resistance after addition of β-lactamase inhibitors, these enzymes may function as a public good, potentially protecting the targeted pathogen as well. Future work should measure β-lactamase activity in predator supernatants to confirm this mechanism. Therefore, the full mechanism of resistance needs to be investigated to understand the potential of piperacillin-based combination therapies.

Thus, in this study, ciprofloxacin was determined as the most promising candidate for combination therapy with *B. bacteriovorus* HD100 against *P. aeruginosa*, as the predator showed high resistance to this antibiotic (MIC 1.306 ± 0.34 μg/mL for 10^6^ PFU/ml and MIC 2.333 ± 0.833 μg/mL for 10^7^ PFU/ml). However, effective therapy requires consideration of *in vivo* antibiotic concentrations. Pharmacokinetic studies of ciprofloxacin have shown maximum plasma concentrations of 2.5 ± 1.1 μg/mL and sputum concentrations of 1.8 ± 1.0 μg/mL during standard oral dosage (Begg et al., 2000). Given that these levels exceed the average MIC values observed for *B. bacteriovorus* HD100 (0.898 ± 0.191 μg/mL), high PFU/ml concentrations of *B. bacteriovorus* may be required to ensure compatibility with standard oral ciprofloxacin dosages. Alternatively, lower ciprofloxacin doses could still be effective when used in combination with *Bdellovibrio.* Finally, introducing plaque-forming predatory bacteria to the human body alongside antibiotics should prompt multiple considerations, such as their predation efficacy in physiologically complex conditions. While its clinical application potential in humans is still unknown, *Bdellovibrio* is non-pathogenic in human serum and epithelial cell cultures (Baker et al., 2017; Gupta et al., 2016; Monnappa et al., 2016; Raghunathan et al., 2019) and induces only mild inflammatory responses in human cell lines (Bonfiglio et al., 2019). Although ineffective in intravenous application (Shatzkes et al., 2016), studies have already shown promising results in this regard for *B. bacteriovorus* against pathogens upon oral administration in animal models (Atterbury et al., 2011; Findlay et al., 2019; Shatzkes et al., 2016; Willis et al., 2016) and in human serum cell lines (Baker et al., 2017; Gupta et al., 2016; Monnappa et al., 2016; Raghunathan et al., 2019), although without antibiotic co-administration. Future research could build on our new assay to investigate combined therapies *in vivo*, offering a deeper understanding of potential clinical applications.

## 5 Conclusion

The here proposed streamlined three-step protocol to measure AMR in plaque-forming predatory bacteria proved effective and robust when using a suitable prey. Our plate-based assay using antibiotic test strips (e.g., E-tests) enables direct readout and removes the need for liquid culture transfers (Figure 2). With this study, our goal was to help lay the groundwork for future clinical applications as the basis for combined treatment studies of antibiotics and plaque-forming predatory bacteria. While promising, these results are preliminary; *in vivo* validation and regulatory studies are necessary.

## Data Availability

The original contributions presented in the study are included in the article/Supplementary material, further inquiries can be directed to the corresponding author.

## Footnotes

^1^ https://www.liofilchem.net/pdf/mic/reading-guide.pdf
