# Supplementary material for: A streamlined method to determine the antibiotic resistance of plaque-forming predatory bacteria
Source: Front Microbiol. 2025 Aug 19;16:1582371. doi: 10.3389/fmicb.2025.1582371 (PMC12401912; doi:10.3389/fmicb.2025.1582371)
Supplement: Supplementary file 1 [file Data_Sheet_1.pdf]

Table S1: Summary of MIC values for tested *Bdellovibrio* strains across independent runs. Columns include the tested antibiotic, *Bdellovibrio* strain, experimental run, technical replicate (trep), exact PFU/ml values, PFU/ml range, and MIC values. MIC statistics are provided as mean MIC, median MIC, standard deviation (SD), and standard error (SE) per run and antibiotic.

Antibiotic	*Bdellovibrio*	run	trep	PFU/ml	PFU/ml_rang	MIC	Mean_MIC	Median_MIC	SD_MIC	SE_MIC
CFZ	HD100	R1	TR1	188666.667	10^5	0.064	0.05333333	0.064	0.01847521	0.01066667
CFZ	HD100	R1	TR2	188666.667	10^5	0.032				
CFZ	HD100	R1	TR3	188666.667	10^5	0.064				
CIP	HD100	R1	TR1	18866.6667	10^4	0.19	0.23	0.25	0.03464102	0.02
CIP	HD100	R1	TR2	18866.6667	10^4	0.25				
CIP	HD100	R1	TR3	18866.6667	10^4	0.25				
CIP	HD100	R1	TR1	188666.667	10^5	0.5	0.46	0.5	0.06928203	0.04
CIP	HD100	R1	TR2	188666.667	10^5	0.5				
CIP	HD100	R1	TR3	188666.667	10^5	0.38				
CN	HD100	R1	TR1	188666.667	10^5	0.064	0.04266667	0.032	0.01847521	0.01066667
CN	HD100	R1	TR2	188666.667	10^5	0.032				
CN	HD100	R1	TR3	188666.667	10^5	0.032				
CIP	HD100	R2	TR1	34800	10^4	0.5	0.58333333	0.5	0.14433757	0.08333333
CIP	HD100	R2	TR2	34800	10^4	0.75				
CIP	HD100	R2	TR3	34800	10^4	0.5				
CIP	HD100	R2	TR1	348000	10^5	0.5	1	1	0.70710678	0.5
CIP	HD100	R2	TR2	348000	10^5	1.5				
CIP	HD100	R2	TR3	348000	10^5	NA				
CN	HD100	R2	TR1	348000	10^5	0.047	0.02633333	0.016	0.01789786	0.01033333
CN	HD100	R2	TR2	348000	10^5	0.016				
CN	HD100	R2	TR3	348000	10^5	0.016				
CFZ	HD100	R3	TR1	2486666.67	10^6	0.032	0.032	0.032	0	0
CFZ	HD100	R3	TR2	2486666.67	10^6	0.032				
CFZ	HD100	R3	TR3	2486666.67	10^6	0.032				
CIP	HD100	R3	TR1	248666.667	10^5	1	0.66666667	0.5	0.28867514	0.16666667
CIP	HD100	R3	TR2	248666.667	10^5	0.5				
CIP	HD100	R3	TR3	248666.667	10^5	0.5				
CIP	HD100	R3	TR1	2486666.67	10^6	1	0.91666667	1	0.14433757	0.08333333
CIP	HD100	R3	TR2	2486666.67	10^6	1				
CIP	HD100	R3	TR3	2486666.67	10^6	0.75				
CN	HD100	R3	TR1	2486666.67	10^6	0.047	0.06266667	0.047	0.02713546	0.01566667
CN	HD100	R3	TR2	2486666.67	10^6	0.047				
CN	HD100	R3	TR3	2486666.67	10^6	0.094				
CFZ	HD100	R4	TR1	1770000	10^6	0.023	0.026	0.023	0.00519615	0.003
CFZ	HD100	R4	TR2	1770000	10^6	0.023				
CFZ	HD100	R4	TR3	1770000	10^6	0.032				
CIP	HD100	R4	TR1	177000	10^5	2	1.5	2	0.8660254	0.5
CIP	HD100	R4	TR2	177000	10^5	0.5				
CIP	HD100	R4	TR3	177000	10^5	2				
CIP	HD100	R4	TR1	1770000	10^6	3	2.5	3	0.8660254	0.5
CIP	HD100	R4	TR2	1770000	10^6	3				
CIP	HD100	R4	TR3	1770000	10^6	1.5				
CN	HD100	R4	TR1	177000	10^5	0.023	0.026	0.023	0.00519615	0.003
CN	HD100	R4	TR2	177000	10^5	0.023				
CN	HD100	R4	TR3	177000	10^5	0.032				
CN	HD100	R4	TR1	1770000	10^6	0.032	0.032	0.032	0	0
CN	HD100	R4	TR2	1770000	10^6	0.032				
CN	HD100	R4	TR3	1770000	10^6	NA				
CFZ	HD100	R5	TR1	1066666.67	10^6	0.023	0.01833333	0.016	0.00404145	0.00233333
CFZ	HD100	R5	TR2	1066666.67	10^6	0.016				
CFZ	HD100	R5	TR3	1066666.67	10^6	0.016				
CFZ	HD100	R5	TR1	10666666.7	10^7	0.125	0.08433333	0.064	0.03521837	0.02033333
CFZ	HD100	R5	TR2	10666666.7	10^7	0.064				
CFZ	HD100	R5	TR3	10666666.7	10^7	0.064				
CIP	HD100	R5	TR1	1066666.67	10^6	0.5	0.5	0.5	0.25	0.14433757
CIP	HD100	R5	TR2	1066666.67	10^6	0.25				

CIP	HD100	R5	TR3	1066666.67	10^6	0.75				
CIP	HD100	R5	TR1	10666666.7	10^7	1.5	2.33333333	1.5	1.44337567	0.83333333
CIP	HD100	R5	TR2	10666666.7	10^7	4				
CIP	HD100	R5	TR3	10666666.7	10^7	1.5				
CN	HD100	R5	TR1	1066666.67	10^6	0.047	0.05833333	0.064	0.00981496	0.00566667
CN	HD100	R5	TR2	1066666.67	10^6	0.064				
CN	HD100	R5	TR3	1066666.67	10^6	0.064				
CN	HD100	R5	TR1	10666666.7	10^7	0.125	0.14666667	0.125	0.03752777	0.02166667
CN	HD100	R5	TR2	10666666.7	10^7	0.19				
CN	HD100	R5	TR3	10666666.7	10^7	0.125				
CFZ	HD100	R6	TR1	1130000	10^6	0.032	0.02366667	0.023	0.00802081	0.00463082
CFZ	HD100	R6	TR2	1130000	10^6	0.023				
CFZ	HD100	R6	TR3	1130000	10^6	0.016				
CFZ	MYbb2	M1	TR1	246000	10^5	0.023	0.02066667	0.023	0.00404145	0.00233333
CFZ	MYbb2	M1	TR2	246000	10^5	0.023				
CFZ	MYbb2	M1	TR3	246000	10^5	0.016				
CFZ	MYbb2	M1	TR1	2460000	10^6	0.032	0.037	0.032	0.00866025	0.005
CFZ	MYbb2	M1	TR2	2460000	10^6	0.032				
CFZ	MYbb2	M1	TR3	2460000	10^6	0.047				
CIP	MYbb2	M1	TR1	246000	10^5	2	1.66666667	1.5	0.28867514	0.16666667
CIP	MYbb2	M1	TR2	246000	10^5	1.5				
CIP	MYbb2	M1	TR3	246000	10^5	1.5				
CN	MYbb2	M1	TR1	246000	10^5	0.032	0.032	0.032	0	0
CN	MYbb2	M1	TR2	246000	10^5	0.032				
CN	MYbb2	M1	TR3	246000	10^5	0.032				
CN	MYbb2	M1	TR1	2460000	10^6	NA	0.0555	0.0555	0.01202082	0.0085
CN	MYbb2	M1	TR2	2460000	10^6	0.047				
CN	MYbb2	M1	TR3	2460000	10^6	0.064				
