# Supplementary material for: A streamlined method to determine the antibiotic resistance of plaque-forming predatory bacteria
Source: Front Microbiol. 2025 Aug 19;16:1582371. doi: 10.3389/fmicb.2025.1582371 (PMC12401912; doi:10.3389/fmicb.2025.1582371)
Supplement: Supplementary file 2 [file Data_Sheet_2.pdf]

Table S2: Statistical analysis of MIC values using a linear mixed model to evaluate the effect of *Bdellovibrio* culture density (PFU/ml) on MIC values. Biological replicates were included as a random factor to account for dependencies among technical replicates performed for the same biological sample. MIC values were log-transformed to ensure normality of residuals, and PFU/ml values were also log-transformed to address the large differences in their magnitudes. Asterisks indicate the p-value for the fixed effect term (PFU/ml): *** < 0.001.

	**Parameter**	**Estimate**	**Std. Error**	**t value**	**df**	**Pr(>|t|)**
Ciprofloxacin	Intercept	-5.92276	1.24881	-4.743	26.22750	6.50e-05 ***
	log(PFU)	0.29951	0.06567	4.561	27.51032	9.54e-05 ***
Gentamicin	Intercept	-10.57547	1.47002	-7.194	17.10131	1.45e-06 ***
	log(PFU)	0.30881	0.07432	4.155	17.11645	0.000654 ***
