# Supplementary figures and images for: A streamlined method to determine the antibiotic resistance of plaque-forming predatory bacteria
Source: Front Microbiol. 2025 Aug 19;16:1582371. doi: 10.3389/fmicb.2025.1582371 (PMC12401912; doi:10.3389/fmicb.2025.1582371)

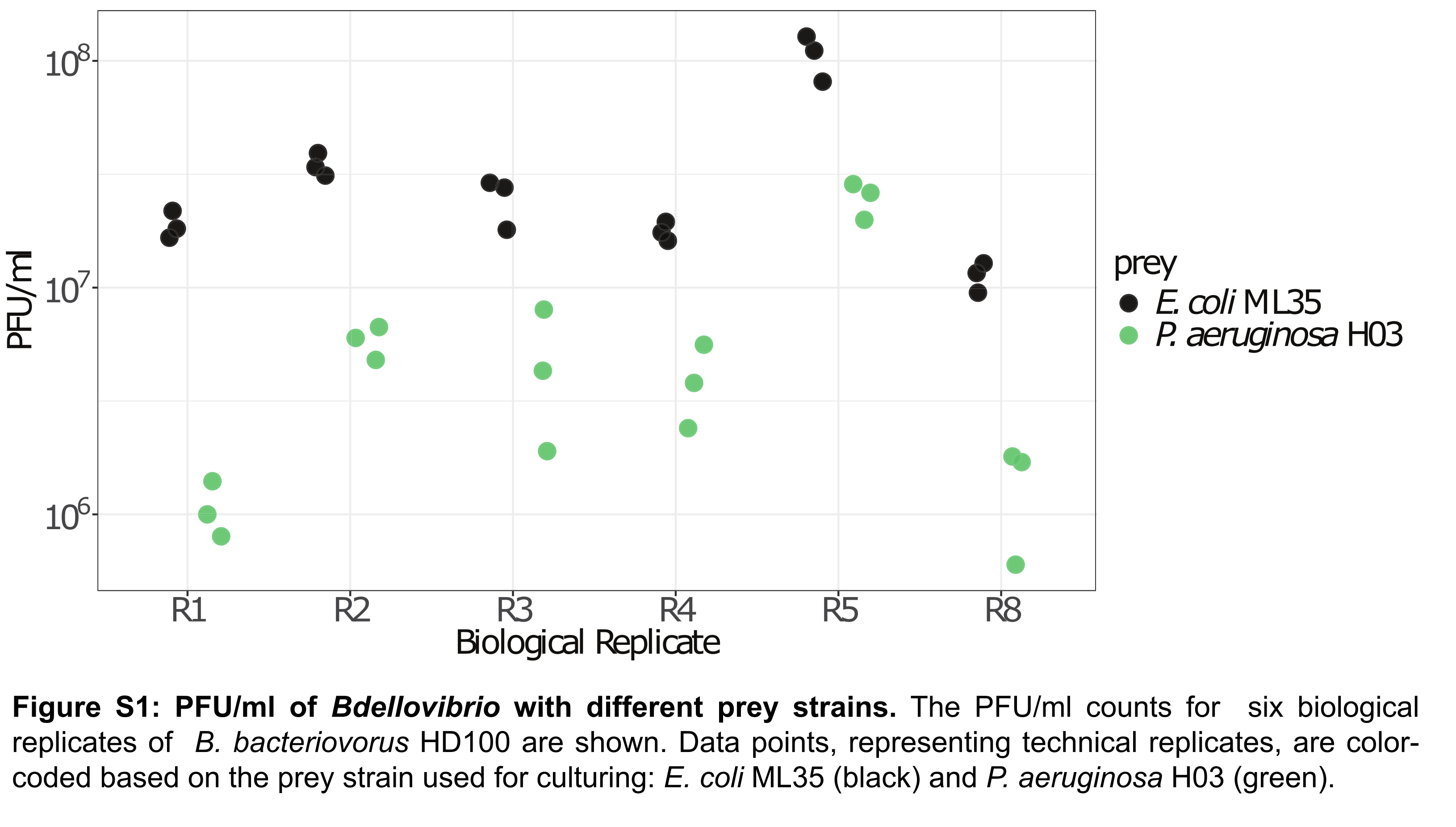

Supplement: Supplementary file 3 [file Image_1.png]

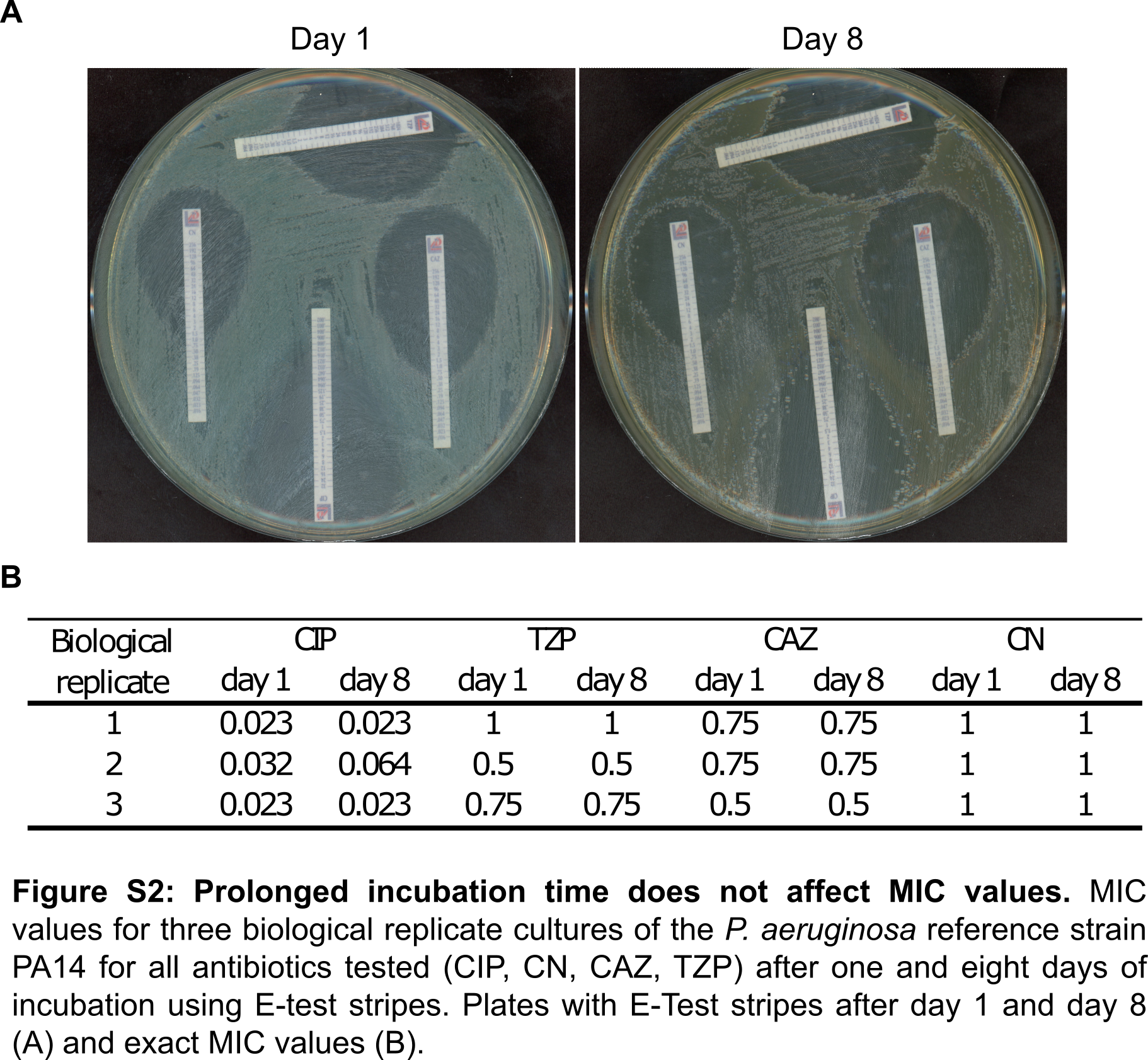

Supplement: Supplementary file 4 [file Image_2.png]

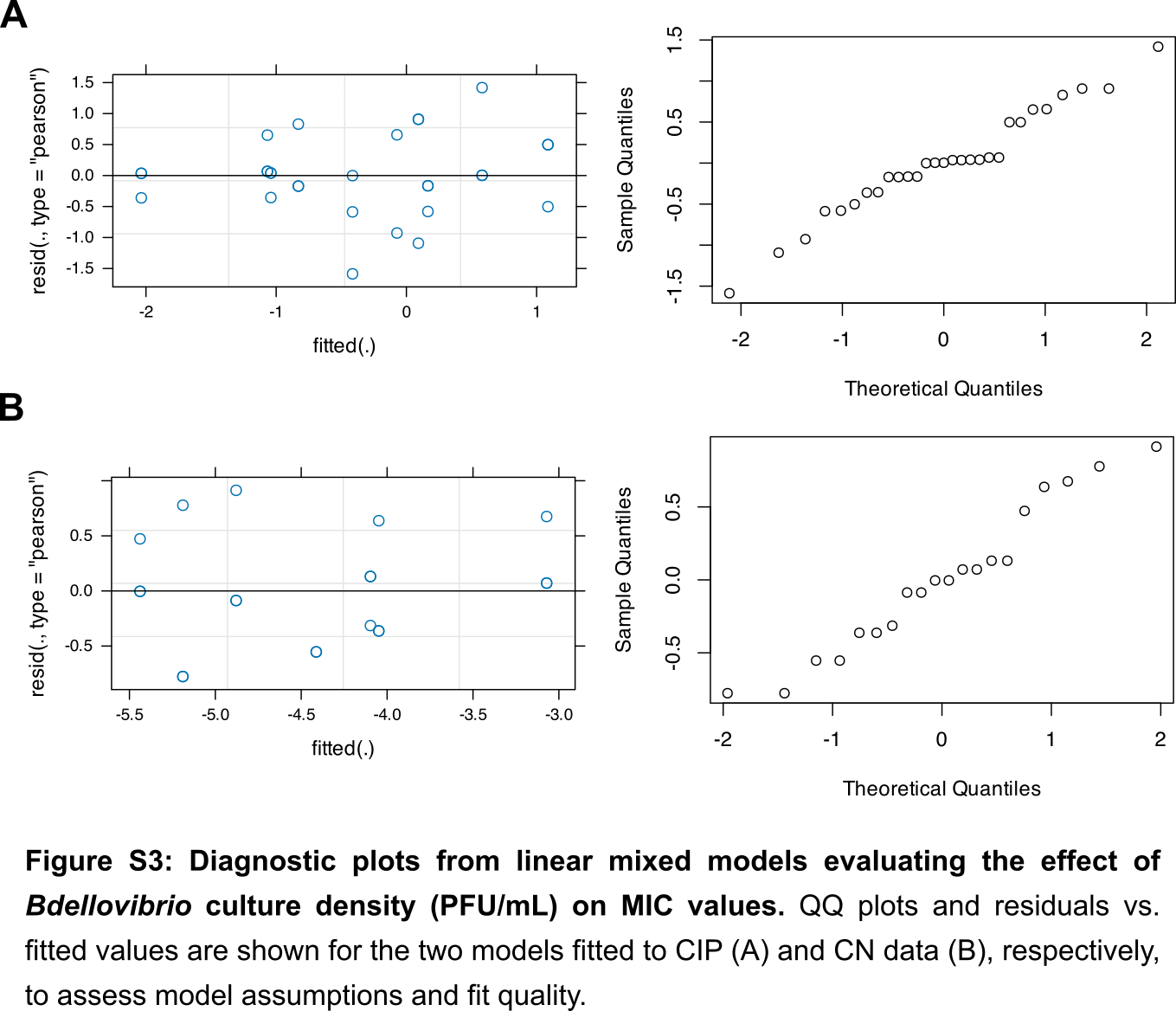

Supplement: Supplementary file 5 [file Image_3.png]
